# Bronchiectasis: Retrospective Analysis of Clinical and Pathological Findings in a Tertiary-Care Hospital

**DOI:** 10.1155/2022/8773204

**Published:** 2022-01-31

**Authors:** Ayse Nur Akatli, Hakkı Ulutas, Emine Turkmen Samdanci, Muhammet Reha Celik

**Affiliations:** ^1^Department of Pathology, Inonu University School of Medicine, Malatya, Turkey; ^2^Department of Thoracic Surgery, Inonu University School of Medicine, Malatya, Turkey

## Abstract

**Background:**

Bronchiectasis is still a challenging chronic lung disease in developing countries. Patients with bronchiectasis can also have pulmonary hypertension. There are sparse data on the prevalence of pulmonary hypertension in patients with bronchiectasis. *Materials and methods*. Archived H&E-stained slides of 141 patients histopathologically diagnosed with bronchiectasis were reevaluated. Cases were categorized into 4 subgroups based on histology: tubular, varicose, follicular, and cystic. In addition, concomitant histopathological changes were also reevaluated. For patients with available CT sections, main, right, and left pulmonary artery (PA) diameters and PA/aorta ratio were measured with regard to pulmonary hypertension.

**Results:**

Of the cases, 70% (*n* = 89) were female and 30% (*n* = 52) were male, with a mean age of 36.58 in females and 33.84 in males. Histopathologically, 43% (*n* = 68) of the cases showed follicular, 37% (*n* = 59) showed varicose, 35% (*n* = 56) showed tubular, and 28% (*n* = 45) showed cystic bronchiectasis morphology. All cases showed chronic inflammation, fibrosis, muscle destruction, and cartilage destruction. *Aspergillus* were present in 11% of the cases showing cystic morphology. Approximately 17% of the cases (*n* = 24) were found to have neuroendocrine cell proliferations. In cases with medial hypertrophy, a statistically significant increase in the left pulmonary artery diameter was radiologically determined.

**Conclusions:**

Medial hypertrophy was found to be significant with regard to indicating a radiological increase in left pulmonary artery diameter. Vascular changes observed in bronchiectasis cases and the presence of neuroendocrine cell proliferations should be specified in pathology reports, and aspergilloma should be carefully investigated in cases with predominant cystic morphology.

## 1. Introduction

Bronchiectasis is still a challenging chronic lung disease in developing countries. It is characterised by permanent dilation and abnormal widening of the bronchi that usually occurs in the context of airway inflammation [1].

The clinical spectrum of the disease is broad. While some patients with mild disease are asymptomatic between exacerbations, others usually have mucoid sputum that becomes purulent during the infective process [1]. The disease can lead to recurrent lower respiratory tract infections, worsening pulmonary functions, respiratory failure, and pulmonary arterial hypertension (PH), resulting in increased morbidity and premature mortality [2, 3].

Patients with bronchiectasis can also have PH and cardiac pathologies [4, 5]. There are sparse data on the prevalence of PH in patients with bronchiectasis. Hence, the clinical importance of PH in bronchiectasis has not yet been completely understood.

In this study, we present the experience of a tertiary-care center regarding the histopathological findings of bronchiectasis, investigated whether histopathological findings could be a useful tool to identify PH in bronchiectasis, and determined which pathological features should be reported in standard bronchiectasis specimens.

## 2. Materials and Methods

**Figure 1:**
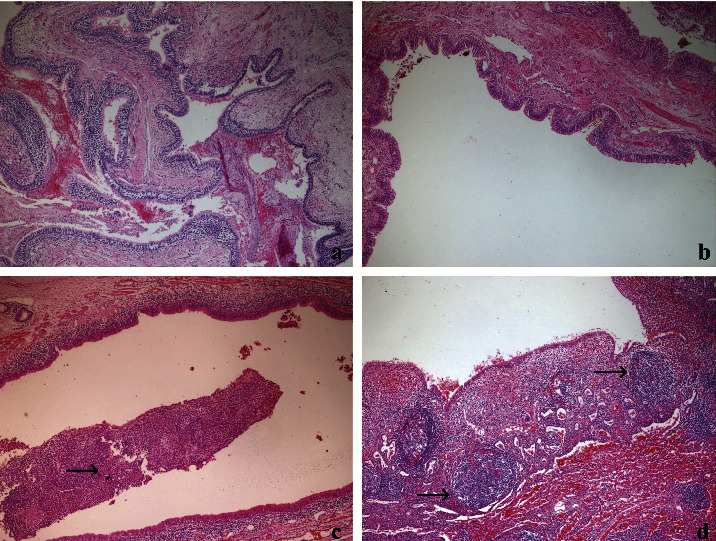
Subtypes of bronchiectasis. (a) Varicose type, (b) cystic type, (c) tubular type, mucous plug in the lumen(arrow), and (d) follicular type, lymphoid follicle formation beneath the epithelium (arrow) (H&E, 40x).

**Figure 2:**
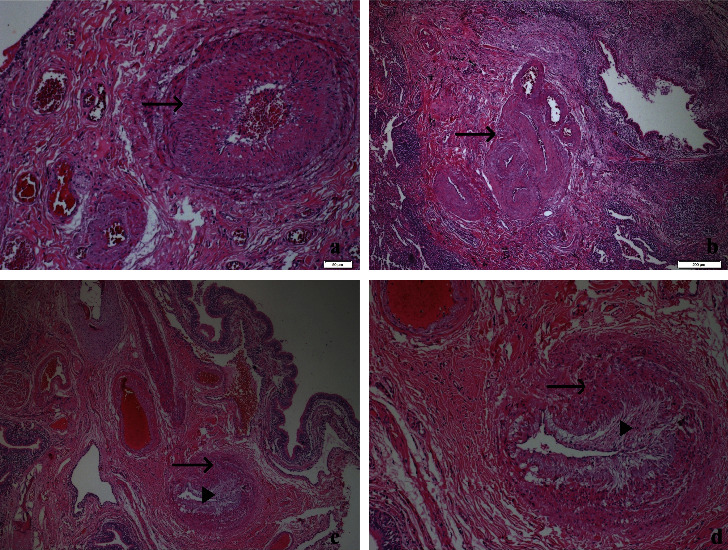
Vascular changes. (a) Medial hypertrophy of an artery (arrow) (H&E, 100x), (b) SiMFi lesion representing hypertrophic anostomoses of vessels (arrow) (H&E, 40x), (c) intimal thickening (arrowhead) and medial hypertrophy (arrow) (H&E, 40x), and (d) intimal thickening (arrowhead) and medial hypertrophy (arrow) on a closer view (H&E, 100x).

**Figure 3:**
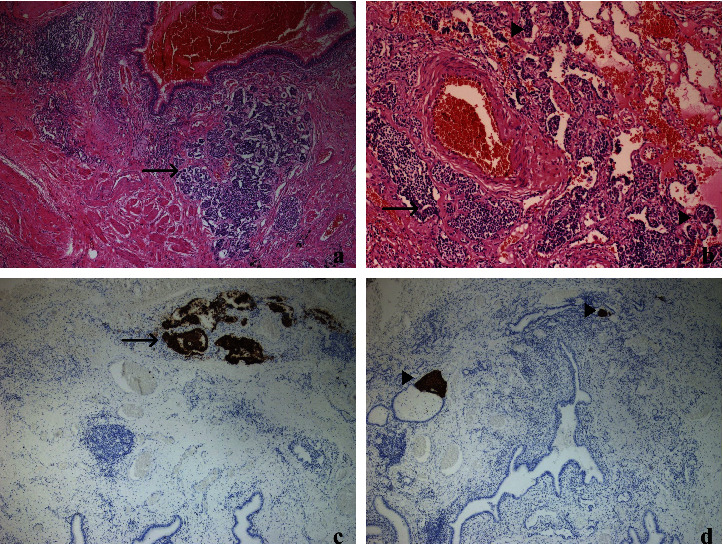
NECH/NET. (a) NET focus (arrow) (H&E, 40x), (b) NECH + NET foci (arrowhead NECH, arrow NET) (H&E, 100x), (c) NET focus positive with chromogranin A (arrow) (40x), and (d) NECH, positive with chromogranin A (arrowhead) (40x).

This is a retrospective study that was approved by the local ethics committee of İnonu University (2020/1121). All procedures performed in the current study were approved by the national research ethics committee (2020/1121) in accordance with the latest version of Helsinki Declaration. This study included 159 pulmonary resection materials of 141 patients who were histopathologically diagnosed with bronchiectasis in the Pathology Laboratory of Inonu University Faculty of Medicine between 1999 and 2019. Archived H&E-stained slides were reevaluated. Cases were categorized into 4 subgroups based on pathology: tubular, varicose, follicular, and cystic [1]. In addition, all slides were reviewed with regard to inflammation and fibrosis on the bronchial wall, disorganized muscle tissue, muscular destruction, cartilage destruction, emphysema, and concomitant changes (such as medial hypertrophy, neuroendocrine cell hyperplasia, tumorlets, interstitial pneumonia, etc.) in the lung parenchyma (Figures 1–3). Medial hypertrophy was evaluated in the wall of distal muscular arteries of <0.5 mm in diameter. The increased thickness of the muscular layer in the media higher than 20% of the vessel's external diameter was accepted to be significant [6]. For patients with medial hypertrophy who have available CT sections, main, right, and left pulmonary artery (PA) diameters and PA/aorta ratio were measured with regard to pulmonary hypertension, using electronic calipers. The CT sections of randomized bronchiectasis patients without medial hypertrophy were used as control groups.

### 2.1. Statistical Analysis

Data were assessed for normal distribution using histograms and Q-Q graphs and the Shapiro–Wilk test. Homogeneity of variance was tested using the Levene test. In cross-group comparisons, the independent two-sample *t*-test was performed for quantitative variables. The comparison of categorical data used Pearson *χ*^2^ analysis. Univariate and multivariate logistic regression analyses (LRA) were performed to determine the risk factors for the groups. Variables with a significance of *p* < 0.05 were introduced to the multiple model, and the backward Wald method was used to determine the independent risk factors. Risk ratios were presented with 95% confidence intervals. Student's *t*-test was used to compare the means of quantitative data obtained by radiology. Data analysis was performed using the TURCOSA (Turcosa Analytics Ltd., Co., Turkey, http://www.turcosa.com.tr) statistics software. The level of significance was accepted as *p* < 0.05.

## 3. Results

Ages of the 141 cases varied between 3 and 68 years with a mean of 35.57 years. Of these cases, 70% (*n* = 89) were female and 30% (*n* = 52) were male, with a mean age of 36.58 in females and 33.84 in males.

Lobectomy was the most common surgical procedure (*n* = 112, 79.43%). Pneumonectomy (*n* = 11, 7,8%), bilobectomy (*n* = 9, 6.38%), and segmentectomy (*n* = 9, 6.38%) were also performed. All patients who underwent pneumonectomy had severe symptoms due to destroyed lungs.

Of the cases, 43% (*n* = 68) were classified as follicular, 37% (*n* = 59) varicose, 35% (*n* = 56) tubular, and 28% (*n* = 45) cystic bronchiectasis. Chronic inflammation, fibrosis, muscular destruction, and cartilage destruction on the bronchial wall were seen in all cases. Of the specimens, 42% (*n* = 59) were right and 58% (*n* = 82) were left lung resection materials, and the most common localization was the lower lobe (57%). The left lower lobe was the most common site of bronchiectatic involvement (42%, *n* = 67). Interstitial pneumonia was determined in 19% (*n* = 31), bronchiolitis obliterans organizing pneumonia (BOOP) in 9% (*n* = 15), and follicular bronchiolitis (FB) in 47% (*n* = 75) of the cases. Neuroendocrine cell hyperplasia and tumorlets (NECH/NET) were seen in 23 cases, and carcinoid tumor was seen in 1 case (Table 1).

Factors influencing the cystic, varicose, and follicular types among the histomorphological subtypes of bronchiectasis were evaluated by single and multiple binary LRAs, while the tubular-type variable was evaluated by single binary LRA. In cases where the number of observations for a variable subgroup was insufficient for analysis, the related variables were not subjected to analysis.

According to the results of single binary LRA, variables that were significant (*p* < 0.05) risk factors influencing the cystic-type bronchiectasis were FB and interstitial pneumonia (Table 2), and risk factors for the varicose-type were FB, bronchopneumonia, medial hypertrophy (MH), and emphysema (Table 3). A significant factor influencing the cystic type was the interstitial pneumonia. *Aspergillus* and bronchopneumonia were determined to be statistically significant in cystic-type bronchiectasis cases, but could not be subjected to LRA due to the insufficient number of cases in the relevant subgroups. Risk factors influencing the varicose type were MH, bronchopneumonia, and emphysematous changes, in descending order of significance (Table 3).

The results of the single and multiple binary LRA for follicular-type bronchiectasis are shown in Table 4. For each unit increase in age, the risk of follicular type was found to decrease 0.97 times (0.95–0.99). On the single binary LRA, the risk factor with a significant effect on the tubular bronchiectasis variable was emphysema (*p* < 0.05).

The only risk factor with a significant effect (*p* < 0.05) on the NECH/NET variable was age.

Variables that were significant (*p* < 0.05) risk factors influencing the MH variable were varicose bronchiectasis, interstitial fibrosis, and age.

We were able to obtain the CT results of 37 of our 45 patients who were determined to have MH, in order to evaluate PH. The CT scans of these cases were compared with the CT scans of 33 randomly selected bronchiectasis cases who did not have MH. There were no statistically significant differences between the two groups with regard to the main PA diameter, PA/aorta diameter, right PA diameter, smoking history, and mean age; however, there was a statistically significant difference between the mean left PA diameters (Table 5).

## 4. Discussion

The purpose of this work was to determine whether any histopathological subtypes of bronchiectasis are correlated with the patients' clinical and morphological characteristics.

Bronchiectasis can be classified according to morphology, anatomy, hemodynamic function, etiology, and clinical presentation. It can be categorized into 4 groups based on histology: tubular (cylindrical), varicose, cystic (saccular), and follicular bronchiectasis [1, 7]. Although these terms were derived from pathological descriptions, they are now mainly used in radiological imaging reports. In tubular bronchiectasis, there is bronchial and bronchiolar dilation in the form of thick tubular structures of uniform appearance that extend toward the lung periphery [1, 8]. In varicose bronchiectasis, the bronchi are irregular in shape and have rounded and bulging ends with alternating areas of dilation and constriction [1]. In cystic bronchiectasis, the bronchi are severely dilated and form large cysts filled with air and fluid [1]. In follicular bronchiectasis, there is excessive lymphoid follicle formation around the bronchi and bronchioles' walls and interstitial pneumonia [9]. However, the clinical usefulness of this classification is controversial, and previous studies could not demonstrate its clinical and pathophysiological significance [10].

With regard to morphological subtypes, we can see that the most common subtype in the present study is the follicular type, followed by varicose, cystic, and tubular types. In cases with the cystic form, we determined FB, *Aspergillus*, bronchopneumonia, and interstitial pneumonia to be more common. We think that, in bronchiectases of cystic morphology, more care must be taken in both clinical and histopathological evaluation, particularly with the opportunistic infections such as *Aspergillus*. On the other hand, we found that bronchopneumonia and emphysema were more likely to be concomitant in the varicose type. The likelihood of MH occurrence was 4.55 was higher in those without the varicose type compared with those with the varicose type.

For the follicular-type bronchiectasis, the variables determined to be significant risk factors were FB, BOOP, bronchopneumonia, interstitial pneumonia, age, and interstitial fibrosis. The notion that all forms of inflammation are found in the follicular type is in agreement with the literature, and it is the form that is most closely associated with infectious agents. A study that investigated the etiological factors of bronchiectasis determined childhood infections in 43% of the cases; however, infection was reported to be the primary cause in 29% of the cases with the disease [3]. We found in the present study that the risk of follicular-type bronchiectasis decreases as age increases and that follicular-type bronchiectasis is particularly prevalent in children and young adults. The varicose, cystic, and tubular bronchiectasis subtypes were not found to have a significant relationship with age.

Neuroendocrine cell hyperplasia, diffuse neuroendocrine cell hyperplasia (DNCH), tumorlets, and typical carcinoid constitute a neuroendocrine tumor group with shared morphological, immunohistochemical, ultrastructural, and molecular characteristics [11]. According to the WHO, the presence of 5 or more neuroendocrine cells distributed in a solitary or cluster pattern in at least 3 bronchioles is defined as neuroendocrine cell hyperplasia if localized within the bronchiolar epithelium or as DNCH if there is diffuse proliferation. If there is a localized infiltrative growth pattern that extends beyond the basement membrane and nodule formation, it is classified based on its size, either as a tumorlet for a nodule diameter <0.5 cm or a carcinoid for a nodule diameter >0.5 cm. Chromogranin A, CD56, and synaptophysin are immunohistochemical markers that are useful in the diagnosis and a low Ki-67 proliferation index (<1%) facilitates differentiation from small-cell carcinoma [12]. In the literature, carcinoid tumor and NETs concomitant with chronic pulmonary diseases were reported mostly in the form of case series [13–16]. The present study also investigated whether or not NECH, NET, and carcinoid tumors seen in bronchiectasis cases had a relationship with the histopathological characteristics. However, no association could be found with any of the histopathological parameters we evaluated, and it was only found that NECH, NET, and carcinoid tumors were encountered more frequently at more advanced ages, consistent with the literature. In agreement with the literature, most of our cases (71%, *n* = 17) were female [17, 18]. Although it has been reported that neuroendocrine cell proliferations can be seen in chronic pulmonary diseases such as bronchiectasis, no data were found in the literature regarding its incidence in bronchiectasis cases. Neuroendocrine cell proliferations were determined in 17% of our cases. This rate corresponds to almost 1/5 of all cases and is certainly not negligible. To the best of our knowledge, this study is the first that shows the prevalence of neuroendocrine cell proliferations in bronchiectasis, and we think that our findings need to be supported by multicenter studies that will involve a larger number of cases.

Although the NETs and carcinoids in our study did not show metastases, lymph node metastases of pulmonary NETs and carcinoids have rarely been reported in the literature [19]. Therefore, the lymph nodes in the macroscopic specimens of the bronchiectasis cases should be obtained with care. Bronchiectatic cases with a diagnosis of NET or carcinoid tumor should not be immediately discharged from clinical follow-up; the possibility of metastasis, although low, should not be disregarded.

Pulmonary hypertension is a common complication encountered in chronic lung diseases [20]. It might be expected in severe and long-term bronchiectasis cases due to hypoxic pulmonary vasoconstriction or remodeling of the vascular bed. However, studies investigating the importance and prevalence of PH in bronchiectasis are quite scarce in the literature because of the fact that tests assessing PH are not routinely performed in bronchiectasis cases [21–24]. There are studies in the literature suggesting that PH seen in cases with severe bronchiectasis can be partially explained by intraparenchymal pulmonary vascular pruning, regardless of its cause [23, 24].

In many nonneoplastic lung diseases such as chronic obstructive pulmonary disease (COPD) and idiopathic pulmonary fibrosis (IPF), intimal thickening and MH are concomitant with mild/moderate PH. On the other hand, conditions associated with endothelial cell proliferative lesions, marked intimal fibrosis, and intimal or medial smooth muscle growth can cause severe PH. Intimal lesions consist of eccentric intimal thickening and fibrotic, plexiform, and concentric angiomatoid lesions. Focal eccentric lesions can be detected in normal lungs, but in PH, these lesions are more widespread [25]. Singular millimetric fibrovascular (SiMFi) lesions have been described recently in lung explants of patients with severe PH [26]. These lesions comprise a large, collagen-rich conglomerate of vessels with a prominent muscular component. SiMFi lesions were more frequently found in lung explants of heritable PH patients, and it is suggested that they may represent the morphologic correlate of anastomoses between pulmonary arteries, bronchial vessels, and pulmonary veins [6]. Medial hypertrophy is one of the characteristic pathological findings of PH. However, smokers may show intimal thickening and MH either with or without PH [25]. Even though MH has an important role in the pathogenesis of PH, linking this finding to PH severity or a specific PA pressure level based on morphology does not yet appear possible [25].

In studies on PH, a main PA diameter >27 mm in females and >29 mm in males on CT were associated with PH. In our series, MH, which is one of the histopathological findings of PH, was determined in the arterial walls of 32% (*n* = 45) of the cases. Upon radiological review of the pulmonary CTs of 37 of these cases, the main PA diameter was determined as 24.00 ± 3.06, as opposed to 22.97 ± 3.00 in cases without MH. In a study conducted by Devaraj et al., PA pressure was reported to have a significant effect on survival based on a radiological increase in the average diameter of right and left PA (>18 mm), while other studies found that main PA diameter was a more significant indicator of PA pressure [21, 27, 28]. There are very few studies on the determination of PH based on CT findings that only included bronchiectasis cases [21]. In terms of the main PA diameter when compared to the study by Devaraj et al., the main PA diameter was found to be lower than 25.26 mm, and the left PA diameter to be equal to 18.69 ± 2.79. Although we did not perform survival analysis; MH was determined to be significant with regard to indicating an increase in left PA diameter. However, it should be noted that there are not many studies related to bronchiectasis.

A PA/aorta diameter ratio >1 was associated with PH in COPD cases [27]. However, Devaraj et al. found a ratio of 0.83, and we have also found a ratio of 0.8. But, it is lower when compared to other studies. The analysis we performed to compare smokers and nonsmokers did not determine any significant differences in PA diameter.

Alzeer et al. and Wang et al. showed that PH was encountered more commonly in cases with cystic bronchiectasis and mixed bronchiectasis [22, 23]. Similarly, we found that the risk of MH occurrence was 4.55 times higher in those without the varicose type compared with the varicose type. This suggests that the morphological subclassification could actually be useful in determining the risk of PH.

The retrospective design of our study and small sample size from a single health-care system is a limitation. However, we think that this study is significant as it highlights the probability of PH in bronchiectasis cases. We think that prospective studies that will involve more cases for the assessment of PH in bronchiectasis cases would be valuable.

## 5. Conclusions

One-third of our cases were histopathologically determined to have MH; although this condition can be present in smokers without PH, we think that it should be considered significant due to indicating increased left PA diameter. It must be remembered that the case should be examined with regard to PH when the pathology report indicates MH. In cases with predominant cystic morphology, aspergilloma should be considered, and the presence of NECH/NET should be specified in the report and lymph nodes in the specimen should be evaluated with diligence.

## Figures and Tables

**Table 1:** Clinicopathological features of cases.

*Gender*	*n*
Female	89
Male	52

*Age*	
Female	36.58
Male	33.84

*Bronchiectasis type*	
Varicose	59
Cystic	45
Tubular	56
Follicular	68

*Accompanying changes*	
FB	75
BOOP	15
Int. pneumonia	31
*Aspergillus*	7
Bronchopneumonia	24
Emphysema	73
Lateralization (right)	59
Lateralization (left)	82
NECH/NET	23
Carcinoid	1
MH	45
Int. fibrosis	55

MH, medial hypertrophy; FB, follicular bronchiolitis; BOOP, bronchiolitis obliterans organizing pneumonia; Int, interstitial; NECH/NET, neuroendocrine cell hyperplasia/neuroendocrine tumorlet.

**Table 2:** Analysis of risk factors that are significant for cystic bronchiectasis.

Variables	Cystic type		*p*	*Binary logistic regression*		*Multiple logistic regression*	
	Absent (*n* = 114)	Present (*n* = 45)		RO (%95 CI)	*p*	RO (%95 CI)	*p*
*FB*							
Present	54(47.4)	30(66.7)	0.043	1.00	—	—	—
Absent	60(52.6)	15(33.3)		2.22(1.08–4.57)	0.030	—	—

*Int. pneumonia*							
Absent	97(85.1)	31(68.9)	0.036	1.00	—	1.00	—
Present	17(14.9)	14(31.1)		2.58(1.14–5.82)	0.023	2.58(1.14–5.82)	0.023

*Aspergillus*							
Absent	112(98.2)	40(88.9)	0.020	—	—	—	—
Present	2(1.8)	5(11.1)		—	—	—	—

*Bronchopneumonia*							
Absent	92(80.7)	43(95.6)	0.035	—	—	—	—
Present	22(19.3)	2(4.4)		—	—	—	—

*Age*	34.25 ± 15.63	35.67 ± 13.72	0.596	1.01(0.98–1.03)	0.594	—	—

Data are expressed as mean *±* standard deviation and (%). RR, risk ratio; CI, confidence interval. FB, follicular bronchiolitis; Int, interstitial; NECH/NET, neuroendocrine cell hyperplasia/neuroendocrine tumorlet (carcinoid is added in the NECH/NET group).

**Table 3:** Analysis of risk factors that are significant for varicose-type bronchiectasis.

Variable	Varicose type		*p*	*Binary logistic regression*		*Logistic regression*	
	Absent (*n* = 100)	Present (*n* = 59)		RR (%95 CI)	*p*	RR (%95 CI)	*p*
*FB*							
Absent	59(59.0)	25(42.4)	0.042	1.00	—	—	—
Present	41(41.0)	34(57.6)		1.96(1.02–3.76)	0.044	—	—

*Bronchopneumonia*							
Absent	92(92.0)	43(72.9)	0.002	1.00	—	1.00	—
Present	8(8.0)	16(27.1)		4.28(1.70–10.77)	0.002	3.69(1.39–9.81)	0.009

*Emphysema*							
Present	53(53.0)	20(33.9)	0.020	1.00	—	1.00	—
Absent	47(47.0)	39(66.1)		2.20(1.13–4.28)	0.021	2.08(1.02–4.25)	0.043

*MH*							
Present	38(38.0)	7(11.9)	0.001	1.00	—	1.00	—
Absent	62(62.0)	52(88.1)		4.55(1.88–11.05)	0.001	4.33(1.74–10.8)	0.002

*Age*	34.69 ± 14.58	34.59 ± 16.02	0.969	1.00(0.98–1.02)	0.969	—	—

Data are expressed as mean *±* standard deviation and (%). RR, risk ratio; CI, confidence interval. MH, medial hypertrophy; FB, follicular bronchiolitis;

**Table 4:** Analysis of risk factors that are significant for follicular-type bronchiectasis.

Variables	Follicular type		*p*	*Binary logistic regression*		*Multiple logistic regression*	
	Absent (*n* = 91)	Present (*n* = 68)		RR (%95 CI)	*p*	RR (%95 CI)	*p*
*FB*							
Absent	63(69.2)	21(30.9)	0.001	1.00	—	1.00	—
Present	28(30.8)	47(69.1)		5.04(2.55–9.94)	<0.001	5.00(2.40–10.43)	<0.001

*BOOP*							
Absent	87(95.6)	57(83.8)	0.025	1.00	—	1.00	—
Present	4(4.4)	11(16.2)		4.20(1.27–13.83)	0.018	5.63(1.47–21.66)	—

*Int. pneumonia*							
Present	23(25.3)	8(11.8)	0.054	1.00	—	—	—
Absent	68(74.7)	60(88.2)		2.54(1.06–6.09)	0.037	—	—

*Aspergillus*							
Absent	84(92.3)	68(100.0)	0.020	—	—	—	—
Present	7(7.7)	0(0)		—	—	—	—

*Bronchopneumonia*							
Absent	84(92.3)	51(75.0)	0.005	1.00	—	—	—
Present	7(7.7)	17(25.0)		4.00(1.55–10.31)	0.004	—	—

*Int. fibrosis*							
Present	39(42.9)	16(23.5)	0.018	1.00	—	1.00	—
Absent	52(57.1)	52(76.5)		2.44(1.21–4.90)	0.012	2.39(1.08–5.30)	0.032

*Age*	37.68 ± 14.60	30.60 ± 14.86	0.003	0.97(0.95–0.99)	0.004	0.96(0.94–0.99)	0.003

Data are expressed as mean *±* standard deviation and (%). RR, risk ratio; CI, confidence interval. FB, follicular bronchiolitis; BOOP, bronchiolitis obliterans organizing pneumonia; Int, interstitial;

**Table 5:** Comparision of patients of brochiectasis with and without MH.

Variables	Group		*p*
	Control (*n* = 33)	MH (*n* = 37)	
Main PA dia.	22.97 ± 3.00	24.00 ± 3.06	0.158
PA/Aort dia.	0.79 ± 0.14	0.80 ± 0.13	0.837
Right PA dia.	18.17 ± 3.33	19.37 ± 3.28	0.134
Left PA dia.	17.23 ± 2.96	18.69 ± 2.79	**0.037**
Smoking	1.06 ± 2.75	5.92 ± 11.01	0.139
Age	36.00 ± 14.81	40.05 ± 13.92	0.242

Data are expressed as mean ± standard deviation. PA: pulmonary artery, Dia.: diameter, MH: medial hypertrophy.

## Data Availability

Data are available on request.

## Additional Points

*What Is Already Known About This Topic.* Patients with bronchiectasis can also have pulmonary hypertension and neuroendocrine cell proliferations.

*What Does This Article Add*. Neuroendocrine cell proliferation incidence in bronchiectasis specimens for the first time in the literature, and we also found out that medial hypertrophy is associated with bronchiectasis subtypes and left pulmonary artery diameters.
